# Comparative efficacy of vitamin B12 supplementation routes in adults with replacement indication: a systematic review

**DOI:** 10.1016/j.eclinm.2026.104214

**Published:** 2026-09-23

**Authors:** Daniela Debone, André Ferreira Azeredo da Silva, Julianne Tannous Cordenonssi, Ricardo V. Cohen

**Affiliations:** a HTAnalyze Consultoria e Treinamento LTDA, Porto Alegre, RS, Brazil; b Institute of Environmental, Chemical and Pharmaceutical Sciences, Federal University of São Paulo, Diadema, SP, Brazil; c Myralis Industria Farmacêutica Ltda, São Paulo, SP, Brazil; d Center for the Treatment of Obesity and Diabetes, Hospital Alemão Oswaldo Cruz, São Paulo, SP, Brazil

**Keywords:** Vitamin B12, Vitamin B12 supplementation, B12 deficiency, Sublingual administration, Intramuscular administration, Oral administration

## Abstract

**Background:**

Vitamin B12 deficiency is common and often requires long-term supplementation to prevent serious complications, yet the comparative efficacy of the oral, sublingual, and intramuscular routes remains uncertain. This systematic review evaluated the efficacy of different vitamin B12 supplementation routes in adults with an indication for replacement.

**Methods:**

PubMed, Embase, and Cochrane CENTRAL were searched without language or date restrictions, from inception to July 18, 2025, updated July 13, 2026, following PRISMA 2020 reporting. Eligible studies were randomized trials and observational cohorts comparing oral, sublingual, or intramuscular vitamin B12 supplementation in adults with an indication for replacement; pediatric and non-comparative studies were excluded. Risk of bias was assessed with RoB 2.0 (trials) and ROBINS-I (cohorts). The primary outcome was serum vitamin B12 or clinical response. This systematic review was registered with PROSPERO, number CRD420251126832.

**Findings:**

13 studies met the inclusion criteria: 7 randomized controlled trials, 3 observational cohorts, 2 conference abstracts, and 1 research letter Included studies were conducted across 10 countries spanning Asia, Europe, North and South America, and Oceania, in both single-center and multicenter settings. Comparisons of oral vs. intramuscular supplementation were most frequent (8 studies), several suggesting comparable or superior biochemical responses with oral administration. Sublingual supplementation was underrepresented (5 studies) but showed short-term biochemical efficacy similar or superior to the other routes. All three routes increased serum vitamin B12 levels. All seven randomized controlled trials were judged to have some concerns regarding risk of bias, mainly due to limitations in randomization procedures and selective outcome reporting; all three observational studies were judged to be at serious risk of bias, primarily due to inadequate confounding adjustment. The remaining three studies (2 conference abstracts, 1 research letter) were not assessed.

**Interpretation:**

Route selection should consider patient preference, tolerability, and access. Evidence for sublingual supplementation remains particularly limited, although the available studies suggest that it may represent a potential non-invasive alternative for selected patients. Future research should include adequately powered comparative studies assessing patient-centered outcomes, such as symptom improvement, treatment adherence, and preference.

**Funding:**

Myralis Industria Farmacêutica Ltda.

**Research in context**

**Evidence before this study**

Before this review, evidence comparing oral, sublingual, and intramuscular vitamin B12 replacement was limited and focused largely on oral vs. intramuscular administration. A 2024 systematic review and network meta-analysis compared all three routes and ranked intramuscular administration highest for biochemical efficacy but found no statistically or clinically significant differences between routes; that review searched only to 2022, was restricted to English-language reports, and pooled adult and pediatric populations within a single analysis. The sublingual route remained supported by few studies, and clinical rather than biochemical outcomes were rarely reported. To provide an up-to-date, adult-focused synthesis with explicit attention to the sublingual route and to clinical outcomes, PubMed, Embase, and Cochrane CENTRAL were searched from inception to July 18, 2025, and the search was updated on July 13, 2026, without language or date restrictions, combining vitamin B12 terms (cobalamin and its forms) with oral, sublingual, and intramuscular route terms (PROSPERO CRD420251126832). Comparative randomized controlled trials and observational cohorts in adults with a replacement indication that reported serum vitamin B12 or clinical response were eligible; non-comparative studies, pediatric populations, reviews, and case reports were excluded. Risk of bias was assessed with RoB 2.0 for trials and ROBINS-I for observational studies. Because of methodological heterogeneity and few head-to-head comparisons, a descriptive synthesis without statistical pooling was performed.

**Added value of this study**

This review adds a prospectively registered (PROSPERO), up-to-date synthesis, searched to July 2026 and unrestricted by language, focused exclusively on adults and thereby avoiding the pooling of pediatric and adult populations present in the earlier network meta-analysis. Across 13 studies, all three routes increased serum vitamin B12, and sublingual administration showed short-term biochemical responses that were generally comparable to those observed with oral and intramuscular administration, although the sublingual evidence base was small (5 studies). No study demonstrated superiority of any route for long-term clinical outcomes, and the certainty of evidence was constrained by open-label designs, unadjusted confounding, and inconsistent outcome reporting, with the observational studies at serious risk of bias. The review corroborates and refines the prior synthesis rather than overturning it, while foregrounding the absence of clinical-outcome data and the underrepresentation of the sublingual route.

**Implications of all the available evidence**

Given that the available evidence suggests broadly similar biochemical responses across the three routes, route selection can reasonably be guided by patient preference, tolerability, and access rather than by presumed differences in efficacy, with non-invasive oral and sublingual options offering practical advantages for adherence. Future research should include adequately powered, long-term randomized trials using standardized, patient-centered clinical outcomes, particularly for the sublingual route, to establish comparative effectiveness.

## Introduction

Vitamin B12, or cobalamin, is essential for numerous metabolic, hematological, and neurological functions. It is indispensable for DNA synthesis, methylation reactions, and myelin formation; it acts as a key cofactor in carboxylic acid conversions that contribute to mitochondrial energy production and is essential for the proper maturation of blood cells. As vitamin B12 cannot be synthesized endogenously, humans must obtain it through dietary intake of animal-derived foods, such as meat, dairy, and eggs, or fortified food products.^1^^,^^2^

Despite its critical physiological roles, vitamin B12 deficiency remains a prevalent nutritional concern worldwide. Its occurrence is influenced by multiple factors affecting intake, absorption, and metabolism,^3^ impacting diverse population subgroups such as children,^4^ people living with diabetes treated with metformin,^5^ individuals who had undergone bariatric surgery,^6^ and vegetarians and vegans. The clinical consequences of vitamin B12 deficiency are diverse and potentially serious, including megaloblastic anemia, peripheral neuropathy, cognitive impairment, and an increased risk of cardiovascular disease.^1^^,^^2^

Vitamin B12 supplementation may be used both to correct established deficiency and for long-term maintenance or prevention in individuals at persistent risk of deficiency. These clinical settings differ in treatment goals, dosing requirements, and expected biochemical responses.^7^

The choice of administration route plays a critical role in the management of vitamin B12 deficiency. Regardless of its etiology, the intramuscular (IM) route is generally recommended in clinical situations involving malabsorption, as it bypasses the gastrointestinal tract, ensuring systemic availability. However, intramuscular injections are associated with some disadvantages, including patient discomfort related to pain, costs due to the need for administration by health care professionals, and the burden of repeated clinic visits.^8^

Although oral supplementation is widely available and has demonstrated efficacy in increasing serum vitamin B12 levels, its use remains controversial in patients with pernicious anemia, a common cause of deficiency characterized by impaired active absorption due to intrinsic factor absence. Historical evidence has shown that approximately 1% of orally ingested vitamin B12 can be absorbed passively, independent of intrinsic factor, supporting the rationale for oral therapy even in malabsorptive conditions. Despite this, the clinical adoption of oral supplementation in pernicious anemia has been limited, largely due to the scarcity of robust, long-term studies specifically targeting this population.^9^

In recent years, alternative delivery methods, including nasal and sublingual formulations, have gained increasing attention. These approaches offer the potential advantages of improved convenience and patient adherence, reducing reliance on healthcare resources. Sublingual administration of vitamin B12 offers physiological advantages due to its rapid absorption through the highly vascularized oral mucosa, directly entering systemic circulation and bypassing hepatic first-pass metabolism. This approach potentially enhances bioavailability and ensures more immediate pharmacokinetic responses compared to intramuscular injections, which require absorption from muscle tissue into the bloodstream.^10^, ^11^, ^12^

Given the potential implications for clinical practice and health policy, a rigorous synthesis of the available evidence comparing sublingual, intramuscular, and oral routes of vitamin B12 supplementation is warranted. Despite growing interest in alternative formulations, primary studies remain limited in both number and methodological robustness. Clarifying the relative effectiveness of these routes is essential to inform evidence-based clinical decisions and to support strategies aimed at improving patient adherence, comfort, and access to care. Serum vitamin B12 concentration is commonly used as a biochemical marker of response to supplementation; however, the magnitude of its increase does not necessarily reflect greater clinical benefit. Accordingly, differences in serum vitamin B12 concentrations between administration routes should not, in isolation, be interpreted as evidence of comparative clinical superiority.^7^ Nevertheless, several studies have compared these administration routes using biochemical and, less frequently, clinical outcomes, supporting the relevance of synthesizing the available evidence and identifying remaining gaps in the literature.

This systematic review aims to provide a descriptive synthesis of the biochemical and clinical outcomes associated with sublingual, intramuscular, and oral vitamin B12 supplementation in patients with a clinical indication for replacement therapy, and to identify gaps in the comparative evidence relevant to clinical practice and future research.

## Methods

This systematic review followed the Cochrane Handbook for Systematic Reviews of Interventions.^13^ Additionally, it was registered in PROSPERO (CRD420251126832, August 20, 2025) and its report was based on the Preferred Reporting Items for Systematic Reviews and Meta-Analyses (PRISMA) statement.^14^

### Eligibility criteria

Scientific articles eligible for inclusion in this systematic review were studies that directly compared the efficacy of different routes of vitamin B12 supplementation in human populations with an established indication for vitamin B12 replacement. The scope of the review was not restricted to maintenance therapy. Eligible studies included adults receiving vitamin B12 supplementation either for treatment of established deficiency or for maintenance or prevention in clinical settings associated with an ongoing indication for replacement. Both randomized controlled trials and observational studies (prospective or retrospective cohorts) were considered. Studies were required to report at least one outcome related to serum vitamin B12 levels or clinical response.

The systematic review primarily included peer-reviewed original articles. Conference abstracts were considered eligible only when they met the predefined inclusion criteria and provided sufficient information for data extraction. Unpublished studies without publicly available results were not included. No language filters were applied in the search strategy to minimize selection bias.

### Inclusion and exclusion criteria

Studies were eligible for inclusion if they were peer-reviewed original articles, designed as randomized controlled trials or observational studies, and conducted in adult patients with a clear indication for vitamin B12 supplementation, such as documented deficiency, malabsorption syndromes, metformin use, or strict vegan diets. Only comparative studies were included, considering any possible comparisons between oral, intramuscular, and sublingual routes of vitamin B12 administration. Eligible studies were required to report outcomes related to efficacy (e.g., changes in serum vitamin B12 levels or clinical improvement).

Exclusion criteria encompassed studies that did not directly compare the administration routes, those that evaluated other routes, such as intranasal administration, studies conducted in pediatric populations, case reports, case series, comments, narrative reviews, systematic reviews, animal studies, in vitro experiments, and articles not available in full text.

### PICO question

Considering the following question: “What is the efficacy of vitamin B12 supplementation routes in patients with an indication for vitamin B12 supplementation?”, the PICO framework was employed to guide the article selection process for inclusion and exclusion and to delineate relevant keywords. The Population (P) included adult patients with a clinical indication for vitamin B12 replacement. The Intervention (I) comprised vitamin B12 supplementation administered through the oral, sublingual, or intramuscular route, and the Comparison (C) comprised direct comparisons between these routes. The Outcomes (O) focused on serum vitamin B12 levels or clinical response as the primary outcome measures.

### Literature search and information sources

A reproducible and comprehensive search was conducted in PubMed, Embase, and Cochrane CENTRAL electronic databases. All relevant synonyms, Medical Subject Headings (MeSH), Emtree terms, and variations of the keywords were considered to ensure broad and sensitive retrieval of eligible scientific articles. The complete search strategies for all databases are provided in Table 1. No filters for publication date or study type were applied. The literature search was initially conducted on July 18, 2025, and was updated on July 13, 2026.

**Table 1:** Search strategy and selection process used in systematic review.

PubMed		
ID	Search strategy	Hits
#1	Search: (“Cobalamin”[MeSH Terms] OR cobalamin[tiab] OR “Cyanocobalamin”[MeSH Terms] OR cyanocobalamin[tiab] OR “Mecobalamin”[Supplementary Concept] OR mecobalamin[tiab] OR “Hydroxocobalamin”[MeSH Terms] OR hydroxocobalamin[tiab] OR “Cobamides”[MeSH Terms] OR cobamides[tiab] OR “Vitamin B 12”[MeSH Terms] OR “vitamin b12”[tiab] OR cobalamins[tiab] OR “Hydroxycobalamin”[MeSH Terms] OR hydroxycobalamin[tiab] OR “hydroxo-cobalamin”[tiab] OR “Vitamin B 12 Coenzymes”[Supplementary Concept] OR “vitamin b12 coenzymes”[tiab] OR “vitamin b 12 coenzymes”[tiab] OR eritron[tiab] OR “b12 vitamin”[tiab] OR “b 12 vitamin”[tiab] OR “vit b12”[tiab] OR “vit b 12”[tiab] OR “vitamin b-12”[tiab] OR vb12[tiab] OR b-12[tiab] OR “Adenosylcobalamin”[Supplementary Concept] OR adenosylcobalamin[tiab] OR “Methylcobalamin”[Supplementary Concept] OR methylcobalamin[tiab] OR mecobe[tiab])	43,673
#2	Search: (“Administration, Sublingual”[MeSH Terms] OR “Sublingual Route”[tiab] OR “Sublingual Administration”[tiab] OR “Sublingual Administrations”[tiab] OR “Drug Administration Sublingual”[tiab] OR “Sublingual Drug Administration”[tiab] OR sublingual[tiab] OR “administered sublingually”[tiab] OR “sublingually administered”[tiab] OR “sublingual formulation”[tiab] OR “sublingual tablet”[tiab])	14,134
#3	Search: (“Administration, Intramuscular”[MeSH Terms] OR “Intramuscular Route”[tiab] OR “Intramuscular Administration”[tiab] OR “Intramuscular Administrations”[tiab] OR “Drug Administration Intramuscular”[tiab] OR “Intramuscular Drug Administration”[tiab] OR intramuscular[tiab] OR “intramuscular formulation”[tiab] OR “intramuscular injection”[tiab])	55,136
#4	Search: (“oral drug administration”[MeSH Terms] OR “oral administration”[tiab] OR “administrations oral”[tiab] OR “oral administrations”[tiab] OR “administration oral drug”[tiab] OR “oral drug administrations”[tiab] OR “oral drug administration”[tiab] OR “drug administration oral”[tiab])	78,831
**#5**	**#1 AND (#2 OR #3 OR #4)**	**666**

EMBASE		
ID	Search strategy	Hits
#1	(‘cobalamin'/exp OR ‘cobalamin':ti,ab OR ‘cyanocobalamin'/exp OR ‘cyanocobalamin':ti,ab OR ‘mecobalamin'/exp OR ‘mecobalamin':ti,ab OR ‘hydroxocobalamin'/exp OR ‘hydroxocobalamin':ti,ab OR 'cobamides'/exp OR 'cobamides':ti,ab OR ‘vitamin b12'/exp OR ‘vitamin b12':ti,ab OR 'cobalamins' OR 'cobalamins':ti,ab OR ‘hydroxycobalamin'/exp OR ‘hydroxycobalamin':ti,ab OR ‘hydroxo-cobalamin':ti,ab OR ‘vitamin b12 coenzymes' OR ‘vitamin b12 coenzymes':ti,ab OR ‘vitamin b 12 coenzymes':ti,ab OR ‘eritron':ti,ab OR ‘b12 vitamin':ti,ab OR ‘b 12 vitamin':ti,ab OR ‘vit b12':ti,ab OR ‘vit b 12':ti,ab OR ‘vitamin b-12':ti,ab OR ‘vb12':ti,ab OR ‘b-12':ti,ab OR ‘adenosylcobalamin'/exp OR ‘adenosylcobalamin':ti,ab OR ‘methylcobalamin'/exp OR ‘methylcobalamin':ti,ab OR ‘mecobe':ti,ab) AND ([embase]/lim OR [medline]/lim)	83,190
#2	(‘sublingual drug administration'/exp OR ‘sublingual route’ OR ‘sublingual administration':ti,ab OR ‘sublingual administrations':ti,ab OR ‘drug administration sublingual':ti,ab OR ‘sublingual drug administration':ti,ab OR ‘sublingual':ti,ab OR ‘sublingual route':ti,ab OR ‘administered sublingually':ti,ab OR ‘sublingually administered':ti,ab OR ‘sublingual formulation':ti,ab OR ‘sublingual tablet':ti,ab) AND ([embase]/lim OR [medline]/lim)	22,299
#3	(‘intramuscular drug administration'/exp OR ‘intramuscular route’ OR ' intramuscular administration':ti,ab OR ' intramuscular administrations':ti,ab OR ‘drug administration intramuscular ':ti,ab OR ' intramuscular drug administration':ti,ab OR ' intramuscular ':ti,ab OR ' intramuscular route':ti,ab OR ' intramuscular formulation':ti,ab) AND ([embase]/lim OR [medline]/lim)	127,224
#4	(‘oral drug administration'/exp OR ‘oral administration':ti,ab OR ‘administrations oral':ti,ab OR ‘oral administrations':ti,ab OR ‘administration oral drug':ti,ab OR ‘oral drug administrations':ti,ab OR ‘oral drug administration':ti,ab OR ‘drug administration oral':ti,ab) AND ([embase]/lim OR [medline]/lim)	518,801
**#5**	**#1 AND (#2 OR #3 OR #4)**	**3492**

Cochrane CENTRAL		
ID	Search strategy	Hits
#1	(“cobalamin” OR “cyanocobalamin” OR “mecobalamin” OR “hydroxocobalamin” OR “cobamides” OR “vitamin b12” OR “cobalamins” OR “hydroxycobalamin” OR “hydroxo-cobalamin” OR “vitamin b12 coenzymes” OR “vitamin b 12 coenzymes” OR “eritron” OR “b12 vitamin” OR “b 12 vitamin” OR “vit b12” OR “vit b 12” OR “vitamin b-12” OR “vb12” OR “b-12” OR “adenosylcobalamin” OR “methylcobalamin” OR “mecobe”)	4929
#2	(“administration sublingual” OR “sublingual route” OR “sublingual administration” OR “sublingual administrations” OR “drug administration sublingual” OR “sublingual drug administration” OR “sublingual” OR “administered sublingually” OR “sublingually administered” OR “sublingual formulation” OR “sublingual tablet”)	5930
#3	(“administration intramuscular” OR “intramuscular route” OR “intramuscular administration” OR “intramuscular administrations” OR “drug administration intramuscular” OR “intramuscular drug administration” OR “intramuscular” OR “intramuscular formulation” OR “intramuscular injection”)	18,324
#4	(“oral drug administration” OR “oral administration” OR “administrations oral” OR “oral administrations” OR “administration oral drug” OR “oral drug administrations” OR “oral drug administration” OR “drug administration oral”)	43,809
**#5**	**#1 AND (#2 OR #3 OR #4)**	**385**

### Study selection

Duplicates were removed using EndNote 20 software (EndNote 20, Thomson Reuters, New York, NY), and titles and abstracts of the records were screened to exclude out-of-scope studies. Subsequently, full texts of the remaining articles were retrieved and reviewed based on the eligibility criteria. Two reviewers independently screened titles and abstracts and subsequently assessed the full-text articles for eligibility. Disagreements at any stage were resolved through consensus. The study selection process was done using Rayyan QCRI software.^15^

### Data collection process and data items

Data was extracted using standardized Excel spreadsheets (Microsoft Corp, Washington, USA) by two reviewers. For each study included in this systematic review, the following data were extracted: authors, year of publication, study design and location, administration routes, population (including participant sex when reported), intervention characteristics, analyzed outcomes, and main findings.

### Risk of bias assessment

The risk of bias of the included studies was assessed independently by two reviewers using appropriate tools according to study design. For randomized controlled trials, the Risk of Bias 2.0 (RoB 2.0) tool developed by the Cochrane Collaboration was used, which evaluates five domains related to the randomization process, deviations from intended interventions, missing outcome data, measurement of the outcome, and selection of the reported result. For non-randomized studies, the Risk of Bias in Non-randomized Studies – of Interventions (ROBINS-I) tool was applied, which assesses bias across seven domains, including confounding, participant selection, classification of interventions, deviations from intended interventions, missing data, measurement of outcomes, and selection of the reported result. Any disagreements over the bias assessment were resolved, if necessary, by discussion and consensus with a third reviewer. The graphical presentation of risk of bias assessments was generated using the RobVis tool (https://www.riskofbias.info/welcome/robvis-visualization-tool). A formal assessment of the certainty of evidence using the GRADE approach was not performed.

### Statistical analysis

A descriptive synthesis was conducted rather than a formal meta-analysis because of the limited number of available studies, the small sample sizes of several studies, and substantial heterogeneity in study design, populations, dosing regimens, duration of follow-up, and outcome reporting, as prespecified in the review protocol. Furthermore, the available evidence was largely concentrated on comparisons between oral and intramuscular supplementation, with few studies specifically addressing the sublingual route. These characteristics were considered to preclude meaningful statistical pooling across studies. As no meta-analysis was performed, statistical heterogeneity measures (e.g., I^2^) and formal assessments of publication bias based on meta-analytic methods, such as funnel plots or Egger’s test, were not applicable. The main characteristics of each study were summarized in tables, including study design, population, intervention details, outcomes assessed, and key findings. Risk of bias assessments were incorporated into the interpretation of results. Effect estimates of serum vitamin B12 levels or clinical response were extracted and presented narratively, without statistical pooling.

### Ethics

This study was a systematic review of previously published studies and data; therefore, ethical approval and informed consent were not required. Ethical conduct of the individual studies included in this review, including adherence to the principles of the Declaration of Helsinki, was the responsibility of the original investigators. The completed PRISMA 2020 checklist is provided in the Supplementary Data (Table S1).

### Role of the funding source

This systematic review was funded by Myralis Indústria Farmacêutica Ltda, São Paulo, Brazil, and was conducted by HTAnalyze Consultoria e Treinamento Ltda under contract to Myralis. The search, study selection, data extraction, and risk-of-bias assessment were performed independently by the HTAnalyze reviewers (DD and AFAS). One author (JTC) is an employee of the funder and contributed to review and editing of the manuscript. The corresponding author had full access to all data and was full responsibility for the decision to submit.

## Results

The literature search retrieved a total of 4543 records. After removing 1499 duplicates, 3044 records remained for title and abstract screening. Of these, 3014 were excluded for not meeting the eligibility criteria. The remaining 30 articles were retrieved in full and assessed for eligibility. 17 studies were excluded at the full-text stage, resulting in 13 studies being included in the final qualitative synthesis. The updated search, performed on July 13, 2026, yielded no additional studies that were eligible for inclusion. The PRISMA flow diagram summarizes the study selection process (Fig. 1).

**Fig. 1:**
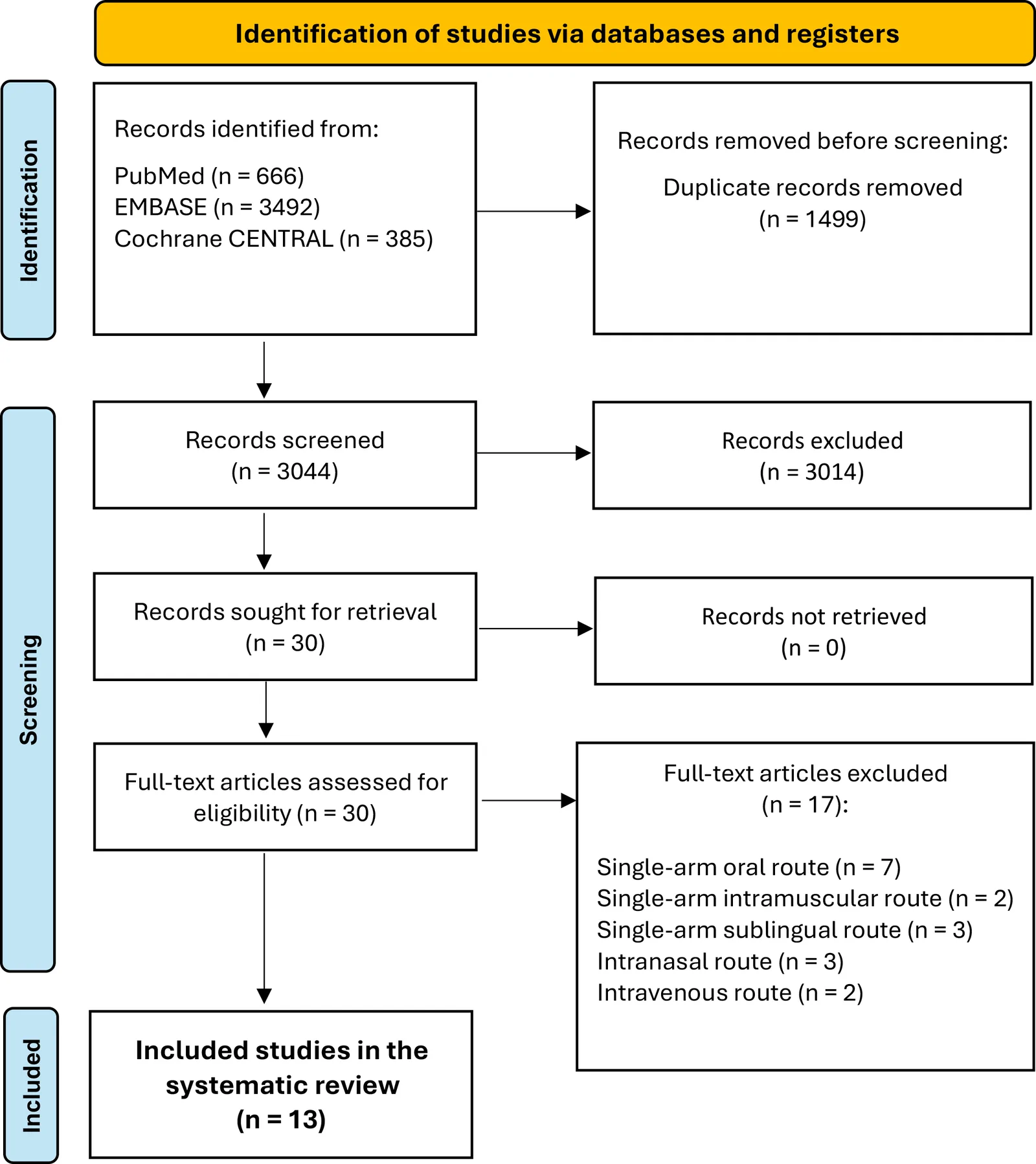
Study profile, according to the Preferred Reporting Items for Systematic Reviews and Meta-Analyses (PRISMA) flow diagram.

Among the 13 studies included in this systematic review, seven were randomized controlled trials, three were observational cohort studies, two were conference abstracts, and one was a research letter. The included studies were conducted in Japan, Israel (n = 2), Turkey, the United States (n = 3), Switzerland, Brazil, Spain, India, Austria, and New Zealand, reflecting a broad geographic distribution across Asia, Europe, the Americas, and Oceania. All were published between 1998 and 2025. Sample sizes varied substantially, ranging from 16 to 4281 participants. Of the included studies, three compared sublingual and intramuscular administration, two evaluated sublingual vs. oral supplementation, and eight assessed the comparative efficacy of oral and intramuscular routes. Although all studies involved adult patients with an indication for vitamin B12 supplementation, their clinical contexts and inclusion criteria differed. Detailed characteristics of the included studies are presented in Table 2.

**Table 2:** Summary of included studies and their main characteristics.

Authors (years)	Study design	Administration routes	Population	Analyzed outcomes	Main findings
Adachi et al. (2000)^16^	Prospective non-randomized clinical study comparing oral and intramuscular VB12 supplementation after total gastrectomy (Japan), between 1991 and 1996.	Intramuscular vs. oral	31 patients who had undergone total gastrectomy (23 men; 8 women). The mean age was 57.2 years (range: 38–74 years.	Serum VB12 concentration; safety.	Both oral and intramuscular vitamin B12 supplementation led to increased serum vitamin B12 levels over 6 months. The study did not perform a statistical comparison between groups. The most frequently reported symptoms were fatigability (39%, 12 of 31), cold feet or legs (32%, 10 of 31), numbness (13%, 4 of 31), and dizziness (13%, 4 of 31). Glossitis, leukoplakia, and erectile dysfunction were each reported in 1 patient (3%).
Bensky et al. (2019)^17^	Retrospective analysis that compared the efficacy of SL vs. the standard IM administration of VB12 in restoring VB12 levels, using computerized Maccabi Healthcare Services pharmacy records, during Jan 2014–Dec 2017 (Israel).	Sublingual vs. intramuscular	4281 patients older than 18 years of age (Sex proportion: not reported), who were prescribed VB12. 19.3% (830 of 4281) were treated with VB12 IM injections and 3451 80.7% (3451 of 4281) with SL tablets. Cohorts with serum VB12 level <300 ng/L include 712 of 830 (86%) patients (IM group) and 2529 of 4281 (73%) patients (SL group). Epidemiological characteristics such as age and gender, as well as possible etiologies for vitamin B12 deficiency, were not evaluated.	Differences between levels of serum VB12 concentration before and after treatment, and hematologic changes: hemoglobin, platelet count, total white blood cell count, and mean cell volume after treatment.	The mean ± SD difference between serum VB12 levels before and after administration of VB12 supplements was significantly higher in the SL group vs. IM injection group (252 ± 223 vs. 218 ± 184 ng/L, p < 0.001). SL VB12 significantly increased the *odds ratio* for increased serum VB12 levels, compared to the IM group, OR 1.85, CI 95% 1.5–2.3, p < 0.001.
Bolaman et al. (2003)^18^	Prospective, randomized, open-label study conducted in patients with megaloblastic anemia due to cobalamin deficiency, between January 1999 and January 2003 (Turkey).	Intramuscular vs. oral	60 adults aged ≥16 years (33 men; 27 women) with megaloblastic anemia secondary to cobalamin deficiency (n = 26 in the oral group and n = 34 in the IM group). 10 were excluded because of failure to appear for follow-up after 10 days of treatment.	Serum levels of vitamin B12 and folate, hematologic parameters, and resolution of neurologic symptoms.	At baseline (Day 0), mean serum vitamin B12 levels were 72.9 ± 54.8 pg/mL in the oral group and 70.2 ± 59.1 pg/mL in the intramuscular group. After 90 days of treatment, VB12 levels increased to 213.8 ± 30.2 in the oral group and 225.5 ± 40.2 in the intramuscular group. No statistical comparison was conducted between the groups; the results suggest comparable efficacy between oral and intramuscular administration.
Castelli et al. (2011)^19^	Prospective, randomized, open-label, parallel-group clinical trial conducted from February 21, 2009, to May 20, 2010, designed to compare the efficacy of oral vs. intramuscular administration of cyanocobalamin in normalizing metabolic markers of vitamin B12 deficiency (United States).	Intramuscular vs. oral	50 healthy patients (11 men; 39 women) with vitamin B12 deficiency, aged ≥18 years; mean (SD) age of 53.2 (15.33) years.	Proportion of patients in each group with normalized serum cobalamin levels (≥350 pg/mL), mean PCFB in serum cobalamin levels, mean PCFB in serum methylmalonic acid, mean PCFB in total plasma homocysteine, mean time to normalization of cobalamin levels, and safety.	Fifty patients were recruited, and 48 (96.0%) completed the study (22 of 24 [91.7%] in the oral VB12 group; 26 of 26 [100%] in the IM group). All patients in both groups achieved serum cobalamin levels ≥350 pg/mL by day 61 and maintained them through day 91. No statistically significant differences were found between groups regarding mean PCFB in serum cobalamin levels (days 61 and 91). No formal statistical analysis was performed for safety outcomes; adverse effects were mostly mild to moderate, and no serious adverse events were deemed related to treatment.
Kuzminski et al. (1998)^20^	Prospective randomized controlled trial conducted in a single center (United States). Patients were randomized to receive either oral or intramuscular VB12 for 4 months, between January 1993 and September 1996.	Intramuscular vs. oral	18 adults with VB12 deficiency (serum VB12 < 200 pg/mL), age 71 ± 11 years (6 men; 12 women). Most had food-cobalamin malabsorption or pernicious anemia.	Serum VB12, methylmalonic acid, homocysteine, hematocrit, reticulocyte count, neurologic symptoms.	After 4 months of treatment, the mean serum VB12 was significantly higher in the oral group (1005 pg/mL) compared to the intramuscular group (325 pg/mL) (p = 0.0005). In patients with cobalamin deficiency, daily administration of 2 mg of oral cyanocobalamin was as effective as monthly intramuscular injections of 1 mg—and may even be superior in achieving higher serum VB12 concentrations.
Metaxas et al. (2017)^21^	Prospective, randomized, nonblinded parallel-group trial that assessed outpatient preferences for VB12 supplementation by the oral or IM route, and to confirm noninferiority of early biomarker response with oral treatment in a typical primary-care population with biochemically defined VB12 deficiency, between November 2013 and December 2015 (Switzerland).	Intramuscular vs. oral	37 patients aged 49.5 ± 18.5 years (14 men; 23 women) were recruited for oral (n = 19) or IM (n = 18) treatment.	Adherence assessed by pill count and dosing irregularities (POEMS); normalization of serum VB12 (>258 pmol/L), holotranscobalamin (>37 pmol/L), homocysteine (<15 μmol/L), and methylmalonic acid (<270 nmol/L); patient preference evaluated at baseline and after 4 weeks via scenario-based questionnaire.	After 28 days, 100% (18 of 18) patients in the IM group achieved normalization of serum VB12 levels, compared to 84.2% (16 of 19) in the oral group; mean VB12 concentrations were 354 pmol/l [95% CI: 298–410] in the oral group and 2796 pmol/l [95% CI: 1277–4314] in the IM group. Before randomization, preference was in favor of oral treatment 45.9% (17 of 37) over IM administration 21.6% (8 of 37). Patients who obtained their preferred route of administration maintained their preference in the case of oral treatment and changed their preference after IM treatment
Ramos et al. (2021)^22^	Prospective controlled cohort study that compared serum vitamin B12 levels in two forms of supplementation (oral vs. intramuscular) for 6 months after gastric bypass, between September 2017 and September 2018 (Brazil).	Intramuscular vs. oral	53 patients were divided into two homogeneous groups: an oral group (n = 24) and an intramuscular group (n = 29), aged 20–60 years (15 men; 38 women).	Serum VB12 and methylmalonic acid concentrations.	Serum vitamin B12 was measured preoperatively and postoperatively at 1, 2, 3, and 6 months. At each point, the serum vitamin B12 level remained within reference values in both groups, although it was higher in the oral group (p < 0.001).
Sanz-Cuesta et al. (2020)^23^	Randomized, multicenter, non-inferiority clinical trial to compare the effectiveness of oral-administered and IM-administered VB12 in the normalization of serum VB12 concentrations at 8, 26 and 52 weeks in patients aged ≥65 years with VB12 deficiency treated at primary healthcare centers, from July 2014 to November 2016 (Spain).	Intramuscular vs. oral	283 patients were allocated to receive VB12 treatment via the IM route (n = 143) or orally (n = 140) (118 men; 165 women); average age of 75.2 (6.34).	Normalization of serum VB12 concentrations (≥211 pg/mL); serum VB12 concentrations (pg/mL); adverse events; adherence to treatment; quality of life (EQ-5D-5L); and patient preferences.	The follow-up period was completed by 80.9% (229 of 283) of patients. At week 8, per-protocol normalization rates were well above 90% in both arms, with no significant difference between oral and IM (133 of 135 vs. 129/130; difference −0.7%, 95% CI −3.2 to 1.8; p = 0.0999). At week 52, normalization by intention-to-treat analysis was 73.6% (103 of 140) in the oral arm and 80.4% (115 of 143) in the IM arm; the per-protocol difference was larger (103 of 112, 92.0%, oral vs. 115 of 117, 98.3%, IM; difference −6.3%, 95% CI −11.9 to −0.07; p = 0.025), while the intention-to-treat difference was smaller and non-significant (difference −6.8%, 95% CI −16.6 to 2.9; p = 0.171). Oral was no less effective than IM at 8 weeks; differences were found between routes at week 52, the probability that these differences exceeded the non-inferiority threshold was low
Sidrar et al. (2024)^24^	Prospective, randomized, open-label controlled trial comparing sublingual and oral vitamin B12 supplementation in adults with vitamin B12 deficiency, from Feb 2022 to Jan 2024 (India).	Sublingual vs. oral	Among 180 randomized adults, 148 completed the study. 72 in the oral group and 76 in the sublingual group (92 men; 56 women). Exclusion criteria included neurological or psychiatric symptoms, renal failure, recent surgery, pregnancy, and gastrointestinal disorders. No age data were reported.	Normalization of serum VB12 level >400 pg/ml.	Both oral and sublingual methylcobalamin (1500 μg/day for 3 months) were equally effective, with normalization of serum VB12 observed in 66.7% (48 of 72) (oral) and 68.4% (52 of 76) (sublingual) of participants (p = 0.82). No adverse events were reported, and compliance exceeded 90% in both groups (absolute numbers not reported).
Shakeri-Leidenmühler (2013)^25^	Prospective, non-randomized, comparative study including two intervention arms for vitamin B12 supplementation in patients after gastric bypass surgery, with follow-up at 3, 6, 9, and 12 months (Austria).	Sublingual vs. intramuscular	Sixteen patients (2 men; (14 women) who underwent gastric bypass surgery, evaluated 7 ± 3 months post-operatively. Participant age was not reported.	Serum VB12 concentration.	Both sublingual and intramuscular supplementation effectively prevented vitamin B12 deficiency over 12 months, with mean serum VB12 levels increasing from 222 ± 55 to 451 ± 321 pmol/L in the sublingual group and from 216 ± 47 to 640 ± 560 pmol/L in the intramuscular group.
Sharabi et al. (2003)^11^	Randomized, controlled, open-label trial comparing sublingual and oral vitamin B12 supplementation in patients with cobalamin deficiency over 8 weeks (Israel).	Sublingual vs. oral	Thirty patients with vitamin B12 deficiency (serum cobalamin <138 pmol/L) without anemia were included. The mean age was 44.5 ± 14.7 years in the sublingual group, 50.2 ± 15.1 years in the oral group, and 49.7 ± 11.6 years in the oral B-complex group. Eighty percent, 70%, and 70% of patients were male in the sublingual B12, oral B12 and oral vitamin B complex groups, respectively (individual group sizes were not explicitly stated).	Serum cobalamin, serum folate, plasma homocysteine, and urine methylmalonic acid concentrations.	Both sublingual and oral regimens significantly increased serum vitamin B12 levels after 4 weeks (p = 0.0001), with no significant difference between routes. Mean levels rose from 94 ± 30 to 288 ± 74 pmol/L (sublingual) and from 108 ± 17 to 286 ± 87 pmol/L (oral) at week 4.
Strong et al. (2016)^26^	RCT that evaluated the effectiveness of sublingual Vitamin B12 supplementation is compared with intramuscular injection for patients using metformin who have low serum vitamin B12. The trial was registered on the Australia New Zealand Clinical Trial registry (ACTRN12612001108808) from Jan 2013 to Feb 2014 (New Zealand)	Sublingual vs. intramuscular	34 T2DM participants were randomized: 19 to the SL tablet, and 15 to the IM injection. The mean (SD) age, duration of diabetes, and duration of metformin use were 64.2 (7.3) years, 13.7 (6.4) years, and 11.6 (5.0) years, respectively (20 men; 14 women).	The primary outcome variable was serum VB12 level after 3 months, adjusted for baseline VB12. The secondary outcomes were the vitamin B12 after 6 months and the Michigan Neuropathy Screening Instrument (MNSI) score after 6 months.	After 3 months, the mean (SD) vitamin B12 was 372.1 (103.3) pmol/L in the SL group compared to 251.7 (106.8) pmol/L in the IM group, ANCOVA test 119.4 (95% CI −191.2 to −47.6), p = 0.002. After 6 months, the mean (SD) serum VB12 was 258.8 (58.7) pmol/L in the SL group and 241.9 (40.1) pmol/L in the IM group (n = 15); ANCOVA test 15.2 (95% CI −50.3 to 19.8), p = 0.38.
Teja et al. (2025)^27^	Retrospective cohort study evaluating vitamin B12 supplementation routes and deficiency surveillance in patients undergoing total gastrectomy between 2014 and 2021 (United States).	Intramuscular vs. oral	52 patients who underwent robotic-assisted total gastrectomy with Roux-en-Y reconstruction; median age of 61 years (range: 29–79). Most patients (43 of 52; 83%) had a diagnosis of gastric cancer, 15% (8 of 52) underwent prophylactic gastrectomy for hereditary gastric cancer syndromes, and 2% (1 of 52) were treated for bleeding familial juvenile polyposis (28 men; 24 women).	Serum VB12 levels, hemoglobin and hematocrit levels; mean corpuscular volume.	43 patients received exclusive oral VB12, while the remaining received parenteral (n = 7) or oral/parenteral (n = 2). At 6-months, 1-year, 2-years and 3-years post-TG, 80% (39 of 49), 68% (30 of 44), 71% (24 of 34) and 50% (8 of 16) of patients, respectively, maintained complete oral supplementation. Mean VB12 levels for oral administration were 1085 (n = 16), 967 (n = 19) and 1012 (n = 18) pg/mL at 6-months, 1-year and 2-years, respectively.

**Notes**. SL: sublingual. IM: intramuscular. VB12: vitamin B12. RCT: randomized controlled trial. T2DM: type 2 diabetes mellitus. SD: standard deviation. OR: *odds ratio*. CI: confidence interval. ANCOVA: analysis of covariance. PCFB: percent change from baseline. EQ-5D-5L: EuroQol 5-Dimension 5-Level questionnaire. MNSI: Michigan Neuropathy Screening Instrument. POEMS: pill count and dosing irregularities. TG: total gastrectomy. *P-values* are as reported by the original studies and reflect the statistical methods of each primary study.

Table 3 summarizes the key characteristics and biochemical outcomes of the included studies. Despite variations in study design, dosing regimens, duration of follow-up, and baseline vitamin B12 levels, all studies reported increases in serum vitamin B12 concentrations following supplementation, regardless of the administration route. Most studies reported short- to medium-term follow-up (ranging from 4 weeks to 24 months), with post-treatment levels generally indicating biochemical response to therapy. However, the magnitude and timing of response varied across routes and formulations. Detailed data on dose, frequency, duration, and changes in serum vitamin B12 concentrations are provided for each study.

**Table 3:** Summary of intervention characteristics and serum vitamin B12 levels before and after treatment across the included studies.

Study	Route	Dose and frequency		Duration	Baseline VB12 levels		Post-treatment VB12 levels	
Adachi et al. (2000)^16^	Intramuscular vs. oral	IM (n = 10) 500 μg/2 months (n = 4) 500 μg/month (n 5 6)	OR (n = 18) 500 μg/day (n = 3) 750 μg/day (n = 10) 1500 μg/day (n = 5)	3 (OR) and 6 (IM) months	IM 102 ± 51 127 ± 51, pg/mL, mean (SD)	OR 132 ± 26 151 ± 25 112 ± 28	IM 328 ± 89 448 ± 329	OR 234 ± 14 417 ± 270 413 ± 142
Bensky et al. (2019)^17^	Sublingual vs. intramuscular	SL (n = 2529) High-dose sub-lingual cyanocobalamin tablet (1 mg) (Contract Pharmacal Corp., NY, USA)/At least 2 packages (each containing 120 tablets) in 6 months	IM (n = 712) High dose of cyanocobalamin ampule (1 mg per mL) (STEROP NV laboratories Inc., Belgium)/1000 μg every other day for 2 weeks, and then once a week for 4 weeks	6 months	SL 226 (41) ng/L, mean (SD)	IM 200 (40.5)	SL 529 (201)	IM 437 (177), p < 0.001
Bolaman et al. (2003)^18^	Intramuscular vs. oral	IM (n = 34) 1000 μg/day of intramuscular cyanocobalamin, injected into the gluteus muscle, for 10 days	OR (n = 26) 1000 μg/day of cyanocobalamin, self-administered orally in fruit juice, for 10 days	3 months (90 days)	IM 70.2 ± 59.1, pg/mL, mean (SD)	OR 72.9 ± 54.8	IM 225.5 ± 40.2	OR 213.8 ± 30.2
Castelli et al. (2011)^19^	Intramuscular vs. oral	IM (n = 26) 1000 μg cyanocobalamin injected IM on days 1, 3, 7, 10, 14, 21, 30, 60, and 90, in fasting state (Commercial sterile cyanocobalamin; NDC: 0517-0031-25)	OR (n = 24) 1000 μg cyanocobalamin tablet daily for 90 days, taken in fasting state with 50 mL water, 1 h before breakfast (Emisphere Technologies, Inc.)	61 and 91 days	IM 262.0 (54.61), pg/mL, mean (SD)	OR 285.5 (54.27)	IM 2258 (1082) (pg/mL, 61d) 2057 (935) pg/mL, 91d) 714.6 (732.11) (%, 61d) 608.9 (446.68) (%, 91d)	OR 1828 (1134) 1957 (947) 452.5 (353.19) 526.9 (350.62)
Kuzminski et al. (1998)^20^	Intramuscular vs. oral	IM (n = 15) 1000 μg of cyanocobalamin intramuscularly on days 1, 3, 7, 10, 14, 21, 30, 60, and 90	OR (n = 18) 2000 μg of oral cyanocobalamin (two 1000 μg tablets; Nature’s Bounty, Bohemia, NY)	4 months	IM 122 ± 35 pg/mL, mean (SD)	OR 133 ± 40	IM 325 ± 165	OR 1005 ± 440, p = 0.0005
Metaxas et al. (2017)^21^	Intramuscular vs. oral	IM (n = 18) Hydroxocobalamin 1000 μg/week IM for 4 weeks (Vitarubin® Depot, Streuli Pharma), mixed with 1% lidocaine	OR (n = 19) Cyanocobalamin 1000 μg/day orally for 28 days (B12 Ankermann®, Wörwag Pharma)	4 visits in 4 weeks	IM V0: 164 (154–174), pmol/L, mean (95% CI)	OR 158 (145–172), p = 0.58	IM V7: 1088 (934–1236) V14: 1897 (1219–2574) V28: 2796 (1277–4314)	OR 304 (250–357), p < 0.001 319 (284–355), p < 0.001 354 (298–410), p < 0.001
Ramos et al. (2021)^22^	Intramuscular vs. oral	IM (n = 29) Once-monthly deep intramuscular injection of a B-complex formulation containing 5000 μg of cyanocobalamin, 100 mg of thiamine, and 100 mg of pyridoxine, starting 30 days after bariatric surgery	OR (n = 24) Daily B-complex multivitamin tablet containing 5000 μg of cyanocobalamin, 100 mg of thiamine, and 100 mg of pyridoxine, starting 30 days after bariatric surgery	6 months	IM 419.2 ± 164.2 pg/mL, mean (SD)	OR 454.1 ± 190.3 p = 0.477	IM 1 mo: 712.9 ± 369.7 2 mo: 555.8 ± 203.9 3 mo: 511.5 ± 276.5 6 mo: 633.4 ± 587.4	OR 689.4 ± 245.5, p = 0.791 1418.2 ± 621.4, p < 0.001 1464.1 ± 536.7, p < 0.001 1484.5 ± 563.5, p < 0.001
Sanz-Cuesta et al. (2020)^23^	Intramuscular vs. oral	IM (n = 143) 1 mg of cyanocobalamin intramuscularly (Optovite®, vial) on alternate days during weeks 1–2, then 1 mg/week during weeks 3–8, and 1 mg/month from week 9–52. Injections were administered at the health center.	OR (n = 140) 1 mg/day of cyanocobalamin orally (Optovite®, vial) for 8 weeks, followed by 1 mg/week up to 52 weeks. Medication was self-administered at home, diluted in water, as instructed.	12 months (8, 26, and 52 weeks)	IM 166.4 (32.6) pg/mL, mean (SD)	OR 173.1 (27.3)	IM vs. OR - % Difference (95% CI) 8 w: PPT –0.7% (−3.2%–1.8%), p = 0.0999 ITT 4.8% (−1.3%–10.9%), p = 0.124 26 w: PPT –12.9% (−17.9% to −6.1%), p < 0.001 ITT–3.2% (−11.8%–5.4%), p = 0.470 52 w: PPT –6.3% (−11.9% to −0.07%), p = 0.025 ITT –6.8% (−16.6%–2.9%), p = 0.171	
Sidrar et al. (2024)^24^	Sublingual vs. oral	SL (n = 76) Sublingual 1500 μg methylcobalamin once daily	OR (n = 72) Oral 1500 μg methylcobalamin once daily	3 months	SL <240 pg/mL	OR <240	SL 68.4% with VB12 > 400 pg/mL	OR 66.7%, with VB12 > 400 pg/mL, p = 0.82
Shakeri-Leidenmühler (2013)^25^	Sublingual vs. intramuscular	SL (n = 9) 500 μg vitamin B12 sublingually every day (Vitamist Spray 4 9 2 puffs)	IM (n = 7) 1000 μg vitamin B12 intramuscularly bimonthly (Erycytol depot 1 mg ampullae)	12 months	SL 222 ± 55, pmol/L, mean (SD)	IM 216 ± 47	SL 3 mo: 384 ± 162 6 mo: 319 ± 172 9 mo: 526 ± 395 12 mo: 451 ± 321	IM 456 ± 281 572 ± 472 414 ± 295 640 ± 560
Sharabi et al. (2003)^11^	Sublingual vs. oral	SL (n = not reported) 500 μg of cyanocobalamin a sublingual tablet (Twinlab, Ronkonkoma, New York, USA)	OR (n = not reported) 500 μg of cyanocobalamin oral tablet (GNC, Greenville, South Carolina, USA) or oral vitamin B complex preparation (two tablets, each containing 250 μg) cobalamin, 100 mg thiamine and 250 mg pyridoxine; Tribemin, ® Bat-Yam, Israel)	8 weeks	SL 94 ± 30 pmol/L, mean (SD)	OR 108 ± 17 98 ± 14	SL 4 w: 288 ± 74 8 w: 279 ± 75	OR 286 ± 87, 293 ± 78 241 ± 73, 266 ± 56
Strong et al. (2016)^26^	Sublingual vs. intramuscular	SL (n = 19) 1 mg/day of SL methylcobalamin in 3 months (Bronson Laboratories, Utah, US)	IM (n = 15) single IM injection of hydroxocobalamin (1 mg) (ABM)	6 months	SL 170.2 (39.0) pmol/L, mean (SD)	IM 166.7 (36.5)	SL 3 mo: 372.1 (103.3) 6 mo: 258.8 (58.7)	IM 251.7 (106.8), p = 0.002 241.9 (40.1), p = 0.38
Teja et al. (2025)^27^	Intramuscular vs. oral	IM (n = 7) The specific formulation, dosage, or frequency is not explicitly detailed in the text Combination - oral + parenteral (n = 2)	OR (n = 43) The specific formulation, dosage, or frequency is not explicitly detailed in the text	24 months	IM Not reported	OR Not reported	IM 6 mo: 848 12 mo: 679 24 mo: 766 pg/mL, mean (SD not reported)	OR 1084 967 1012

SL: sublingual; IM: intramuscular; OR: oral; VB12: vitamin B12; CI: confidence interval; SD: standard deviation; mo: month(s); w: week(s); d: day(s); V: visit; ITT: intention-to-treat; PPT: per-protocol treatment.

The included studies employed a wide variety of dosages and administration schedules across all routes. Oral and sublingual supplementation were administered once daily in most studies, whereas intramuscular protocols ranged from single injections to repeated administrations over extended periods.

Biochemical outcomes were reported heterogeneously across studies. While some presented mean serum vitamin B12 concentrations, others reported categorical thresholds, such as the proportion of patients reaching specific target levels. There was no consistent standard for defining biochemical response, and units of measurement varied between pg/mL and pmol/L. Moreover, several studies lacked p-values, confidence intervals, or robust statistical analyses, limiting the ability to perform quantitative comparisons of efficacy across administration routes.

Among the studies comparing oral and intramuscular supplementation, Ramos et al. (2021)^22^ and Kuzminski et al. (1998)^20^ reported more favorable biochemical responses with the oral route, while Metaxas et al. (2017)^21^ found greater increases in serum VB12 levels following intramuscular administration. Sanz-Cuesta et al. (2020)^23^ reported similar outcomes between the two routes in the intention-to-treat analysis; although the per-protocol analysis slightly favored the intramuscular group, the findings suggest non-inferiority of the oral route.

Regarding the sublingual route, Shakeri-Leidenmühler et al. (2013),^25^ Strong et al. (2016),^26^ and Bensky et al. (2019)^17^ compared sublingual with intramuscular supplementation and reported comparable or superior short-term biochemical responses with the sublingual route. Additionally, two studies^11^^,^^24^ compared sublingual and oral administration, finding no significant differences in post-treatment vitamin B12 levels.

Compared with oral and intramuscular routes, the sublingual route was underrepresented, with only five studies available and limited comparative data. The evidence illustrates considerable variability in outcomes across studies and underscores the need for cautious interpretation. Overall, the studies reported increases in serum vitamin B12 concentrations following supplementation, regardless of the administration route.

Four studies reported data on patient preference or acceptability. In Sanz-Cuesta et al., (2020),^23^ 83.4% (236 of 283) of participants preferred oral administration, including 97.6% (136 of 140) of those receiving oral vitamin B12 and 68.6% (98 of 143) of those receiving intramuscular treatment; the between-group difference was 29.0% (95% CI 20.3–37.7; p < 0.001). Metaxas et al., (2017)^21^ reported that, before randomization, 45.9% (17 of 37) of participants preferred oral treatment, 21.6% (8 of 37) preferred intramuscular treatment, and 32.4% (12 of 37) had no preference. Preferences were influenced by concerns related to pain, inconvenience, time consumption, and treatment adherence, and 24.3% (9 of 37) of participants changed their preference after treatment. In Shakeri-Leidenmühler (2013),^25^ among patients receiving sublingual vitamin B12 spray, 78% (7 of 9) were dissatisfied with the high frequency of administration, although 67% (6 of 9) reported that they would recommend the treatment. Teja et al., (2025)^27^ reported that oral supplementation was the predominant route used after total gastrectomy, whereas smaller proportions of patients preferred intramuscular or subcutaneous administration.

Regarding risk of bias assessment, randomized controlled trials were evaluated using the Cochrane Risk of Bias 2.0 (RoB 2.0) tool, while observational studies were assessed with the Risk of Bias in Non-Randomized Studies of Interventions (ROBINS-I) tool, allowing for a structured appraisal of methodological quality and potential sources of bias (Figs. 2 and 3).

**Fig. 2:**
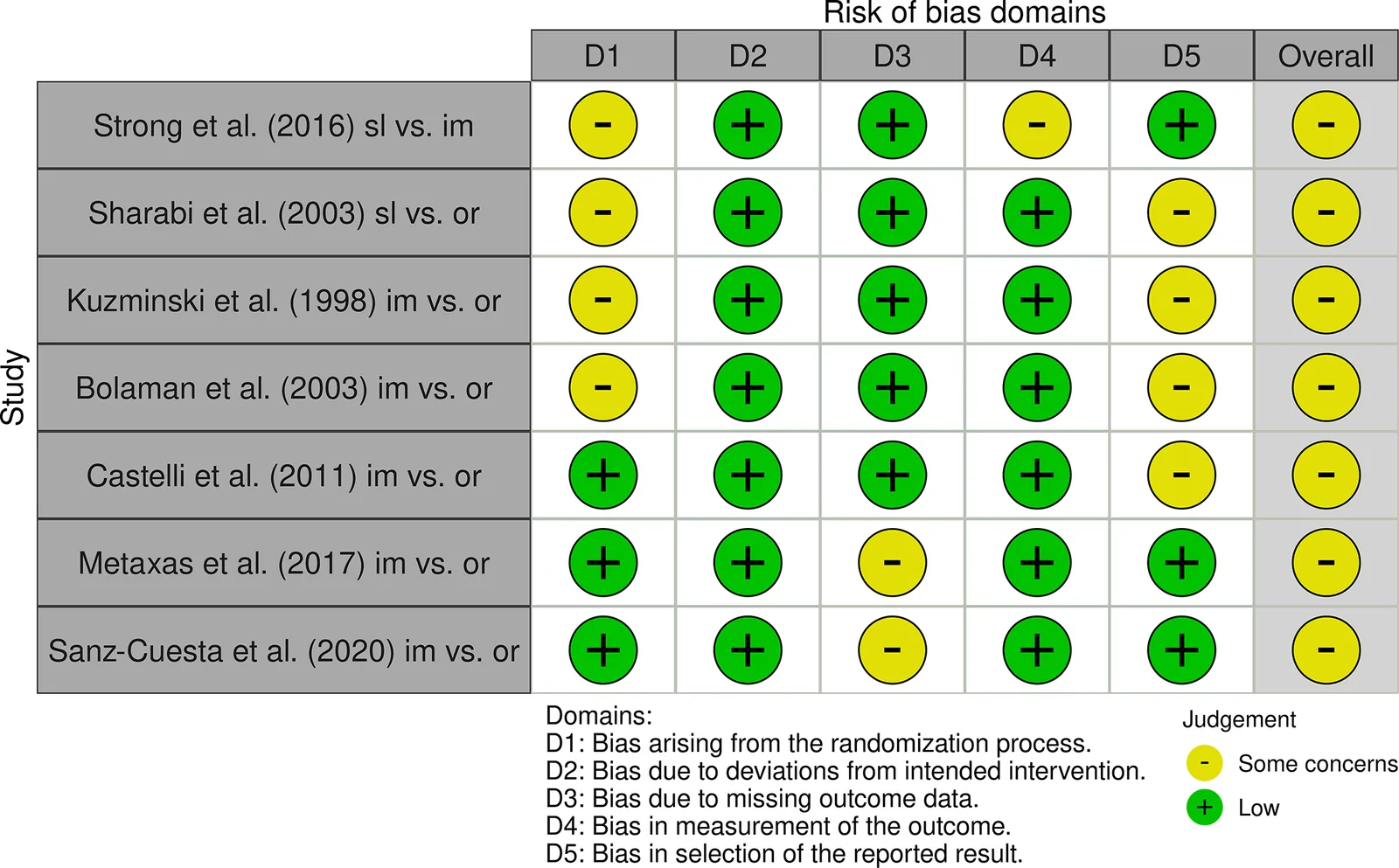
Risk of bias assessment of the included randomized controlled trials using the Cochrane Risk of Bias 2.0 (RoB 2.0) tool.

**Fig. 3:**
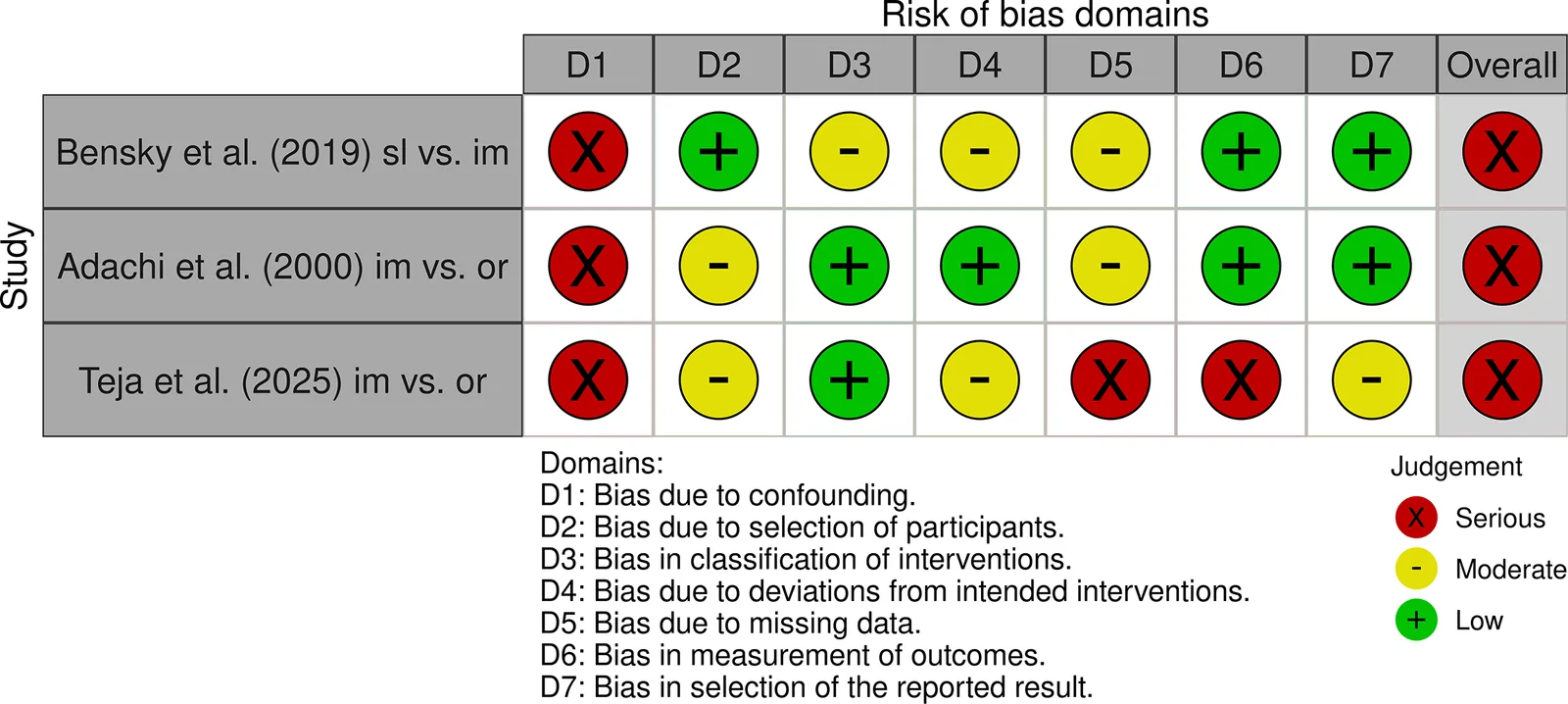
Risk of bias assessment of the included observational studies using the Risk of Bias in Non-Randomized Studies of Interventions (ROBINS-I) tool.

In Domain 1 (bias arising from the randomization process), four studies were rated as having “some concerns” due to insufficient information on sequence generation and/or allocation concealment, limiting the ability to assess the robustness of the randomization procedures. In Domain 5 (bias in selection of the reported result), four studies were similarly penalized, mainly because of the lack of formal statistical analyses or incomplete reporting of outcome data, which hindered the accurate interpretation of the results. Overall, the included randomized controlled trials were judged as having “some concerns” regarding, reflecting methodological limitations that affect the certainty of the evidence across studies (Fig. 2).

Regarding observational studies, in Domain 1 (bias due to confounding), all three observational studies were rated as having serious risk of bias, reflecting the absence of appropriate adjustment for potential confounders, such as baseline clinical severity, and comorbidities. In Domain 5 (bias due to missing data), one study was rated as having serious risk and another as moderate. These judgments were based on multiple limitations, including lack of information on sample losses, subgroup analyses reported with limited transparency, absence of sensitivity analyses, and substantial attrition in follow-up data over time, compromising longitudinal interpretation. In Domain 6 (bias in measurement of outcomes), one study was penalized despite the use of objective laboratory values, due to unclear statistical methodology: no formal comparisons between supplementation groups were reported, and p-values were not presented, reducing the clarity and interpretability of the findings. Overall, all observational studies were judged to have a serious risk of bias, which limits the certainty of the evidence derived from these sources (Fig. 3). Shakeri-Leidenmühler et al. (2013),^25^ Sidrar et al. (2024),^24^ and Ramos et al. (2021)^22^ were not included in the risk of bias assessment, as they were published as conference abstracts or a research letter.

## Discussion

This systematic review aimed to assess the comparative efficacy of sublingual, oral, and intramuscular vitamin B12 supplementation in adults with a clinical indication for replacement therapy. Despite heterogeneity across studies, the findings consistently demonstrated that all three routes were associated with increases in serum vitamin B12 levels, suggesting biochemical efficacy irrespective of the mode of administration. A total of 13 studies were included: seven were randomized controlled trials, three were observational cohort studies, two were conference abstracts, and one was a research letter. Comparisons between oral and intramuscular routes were the most frequently reported (eight studies), whereas sublingual supplementation was comparatively underrepresented in the literature. Only three studies compared sublingual and intramuscular administration, and two evaluated sublingual vs. oral supplementation. This imbalance in the available evidence limits the strength of conclusions regarding the relative efficacy of the sublingual route.

Among the studies comparing sublingual and intramuscular supplementation, biochemical responses were generally comparable, with some evidence favoring the sublingual route in the short term. In the 12-month study by Shakeri-Leidenmühler et al. (2013),^25^ in the context of bypass surgery, both routes effectively prevented vitamin B12 deficiency, with mean serum VB12 levels increasing from 222 ± 55 to 451 ± 321 pmol/L in the sublingual group and from 216 ± 47 to 640 ± 560 pmol/L in the intramuscular group. Strong et al. (2016)^26^ reported a significantly greater biochemical response in the sublingual group of patients with T2DM after three months (372.1 [103.3] pmol/L in the SL group vs. 251.7 [106.8] pmol/L in the IM group; ANCOVA test 119.4 [95% CI –191.2 to −47.6], p = 0.002), although this difference was no longer significant at six months (258.8 [58.7] vs. 241.9 [40.1] pmol/L; p = 0.38). Similarly, Bensky et al. (2019),^17^ in a large retrospective cohort, observed significantly greater mean increases in serum VB12 levels in the sublingual group compared to the intramuscular group (252 ± 223 vs. 218 ± 184 ng/L, p < 0.001), as well as higher odds of achieving increased VB12 levels (OR 1.85; 95% CI 1.5–2.3; p < 0.001). These findings suggest that sublingual supplementation may offer a non-inferior, and in some cases superior, biochemical response compared to intramuscular administration in the short-term.

Two studies compared sublingual and oral vitamin B12 supplementation and found no significant differences in biochemical response between the routes. In the study by Sharabi et al. (2003),^11^ both regimens led to significant increases in serum VB12 levels after four weeks (p = 0.0001), with mean concentrations rising from 94 ± 30 to 288 ± 74 pmol/L in the sublingual group and from 108 ± 17 to 286 ± 87 pmol/L in the oral group. Similarly, Sidrar et al. (2024)^24^ reported that daily administration of 1500 μg methylcobalamin for three months resulted in serum VB12 normalization in 68.4% (52 of 76) of participants in the sublingual group and 66.7% (48 of 72) in the oral group (p = 0.82). No adverse events were observed, and treatment compliance exceeded 90% (absolute numbers not reported) in both groups. These findings suggest that both oral and sublingual routes can be effective in restoring serum vitamin B12 levels in the short term, although differences in dosing regimens between studies limit direct comparisons of their relative efficacy.

Among the studies comparing oral and intramuscular supplementation, findings were mixed supporting the biochemical efficacy of both routes. Kuzminski et al. (1998)^20^ reported significantly higher serum VB12 levels in the oral group (1005 pg/mL) compared to the intramuscular group (325 pg/mL) after four months of treatment (p = 0.0005), suggesting that daily oral administration of 2 mg cyanocobalamin may be as effective—or even superior—to monthly intramuscular injections. In contrast, Metaxas et al. (2017)^21^ found greater increases in serum VB12 concentrations with intramuscular administration over 28 days, with 100% (18 of 18) of patients in the IM group achieving normal levels, compared to 84.2% (16 of 19) in the oral group; mean concentrations were 2796 pmol/l [95% CI: 1277–4314] in the IM group vs. 354 pmol/l [95% CI: 298–410] in the oral group. Interestingly, although most participants initially preferred oral supplementation, patient preference shifted in favor of the IM route after treatment in the IM group. In the 12-month randomized trial by Sanz-Cuesta et al. (2020),^23^ both routes achieved high rates of VB12 normalization at week 8 by per-protocol analysis (98.5% [133 of 135] oral vs. 99.2% [129 of 130] IM; difference −0.7%, 95% CI −3.2 to 1.8), with small, non-significant differences also observed in the intention-to-treat analysis; the per-protocol analysis showed slightly better outcomes for the IM route at week 52 (92.0% [103 of 112] oral vs. 98.3% [115 of 117] IM; difference −6.3%, 95% CI −11.9 to −0.07). Under a Bayesian framework, the probability of non-inferiority of oral supplementation (Δ > −10%) at week 52 was low (0.036 per-protocol; 0.060 intention-to-treat), indicating that non-inferiority of the oral route was not established at this timepoint. Ramos et al. (2021),^22^ in a cohort of patients who had undergone gastric bypass surgery, found that serum VB12 remained within normal limits in both groups, although levels were consistently higher in the oral group across all time points, including a significant difference at six months (1484.5 ± 563.5 vs. 633.4 ± 587.4 pg/mL, p < 0.001). Taken together, these findings support the use of oral supplementation as a viable alternative to intramuscular administration, particularly in contexts of self-administration, cost, or adherence may influence clinical decision-making. These differences in post-treatment serum vitamin B12 concentrations should be interpreted cautiously, as higher circulating levels following pharmacological supplementation do not necessarily indicate greater clinical effectiveness or a clinically meaningful advantage of one administration route over another.

In addition to biochemical efficacy, few studies reported on safety outcomes and patient-reported measures associated with oral and intramuscular supplementation. Adachi et al. (2000),^16^ in a cohort of patients who had undergone total gastrectomy, observed improvements in serum VB12 levels with both routes over six months, but did not perform statistical comparisons between groups. A range of symptoms was reported, such as fatigability (39%, 12 of 31), cold feet or legs (32%, 10 of 31), numbness (13%, 4 of 31), and dizziness (13%, 4 of 31). Glossitis, leukoplakia, and erectile dysfunction were each reported in 1 patient (3%), although their relationship to treatment was not clearly defined. Castelli et al. (2011)^19^ found that all patients achieved and maintained serum cobalamin levels ≥350 pg/mL by day 91, with no significant differences in biochemical response between groups. While adverse events were not formally analyzed, they were mostly mild to moderate, and no serious events were attributed to the intervention. In the trial by Sanz-Cuesta et al. (2020),^23^ adverse events were infrequent and evenly distributed across treatment arms (5 of 11 events, 3.57%, oral vs. 6 of 11 events, 4.20%, intramuscular; p = 0.786), and quality-of-life measures were comparable. Notably, a large majority of patients (83.4%; 236 of 283) expressed a preference for the oral route. These findings support the acceptability and tolerability of oral supplementation, which may be an important consideration in clinical decision-making, particularly in long-term replacement strategies.

Beyond efficacy in improving serum vitamin B12 levels, the favorable outcomes associated with sublingual administration reflect both its pharmacological efficacy and greater practicality in clinical settings. Unlike intramuscular therapy, which depends on healthcare access and professional administration, sublingual formulations can be self-administered, potentially enhancing adherence, increasing patient comfort, and contributing to better quality of life. The sublingual route may also be particularly advantageous for patients with absorption impairments, offering a non-invasive alternative that does not rely on gastrointestinal uptake.^8^^,^^28^ Nevertheless, the limited number of studies evaluating sublingual supplementation restricts the robustness of these observations. The imbalance in the available evidence limits the strength of conclusions regarding the relative efficacy of the sublingual route, underscoring the need for cautious interpretation.

Taken together, our findings reinforce the biochemical effectiveness of vitamin B12 supplementation across oral, intramuscular, and sublingual routes. Despite variations in study design, dosing regimens, duration of follow-up, and baseline vitamin B12 levels, all studies consistently reported increases in serum vitamin B12 concentrations following supplementation. These results are broadly in line with those of a 2024 systematic review.^28^ Although the conclusions align, this review differs from that synthesis in several respects that strengthen its applicability. First, it was prospectively registered on PROSPERO, whereas the earlier review was not registered. Second, the search was current to July 2026, approximately 4 years more recent, and captured studies published after the earlier search, including a 2024 sublingual-vs.-oral trial and a 2025 total-gastrectomy cohort. Third, no language restriction was applied, in contrast to the English-only filter of the earlier review. Fourth, and most consequentially, eligibility was restricted to adults with a clinical indication for replacement, avoiding the pooling of adult and pediatric populations, including infants and school-aged children, that limited the comparability of the earlier network meta-analysis. Finally, this review incorporated conference abstracts and a research letter that the earlier review excluded, and it places explicit emphasis on the scarcity of long-term clinical outcomes and on the underrepresented sublingual route. These differences explain the partial overlap in included studies and position the present review as a registered, current, and adult-focused synthesis that corroborates and refines, rather than replaces, the earlier quantitative work.

This systematic review has several limitations. First, the limited number of available studies, the small sample sizes of several included studies, and the heterogeneity in study design, populations, dosing regimens, duration of follow-up, and outcome reporting precluded a formal meta-analysis and limited the comparability of results. Second, many included studies had methodological weaknesses, such as open-label designs, lack of robust statistical analyses, or insufficient adjustment for confounding, which may introduce bias. Third, the available evidence was largely based on biochemical outcomes, particularly changes in serum vitamin B12 levels, with limited assessment of clinically meaningful outcomes. In addition, the relative scarcity of studies evaluating sublingual supplementation, particularly in head-to-head comparisons, reduces the strength of conclusions regarding this route. Finally, although no language restrictions were applied in the search strategy, the possibility of publication bias remains, and some relevant data may not have been captured. The descriptive nature of this synthesis precluded a subgroup analysis to assess whether efficacy varied by sex. These limitations should be considered when interpreting the findings and their implications for clinical practice: although the current literature supports the biochemical efficacy of vitamin B12 supplementation across routes, the overall strength of this conclusion is moderate, and its applicability to clinical decision-making should be approached with caution.

In conclusion, this systematic review indicates that sublingual, intramuscular, and oral vitamin B12 supplementation can improve serum VB12 levels in adults with a clinical indication for replacement therapy. However, the available evidence is limited and heterogeneous and is largely based on biochemical outcomes rather than clinically meaningful endpoints. Evidence for sublingual supplementation remains particularly limited, although the available studies suggest that it may represent a potential non-invasive alternative for selected patients. The safety profile of sublingual supplementation remains unclear, as none of the included studies reported adverse events for this route, and safety data were limited for oral and intramuscular administration as well. Most of the available evidence focused on comparisons between intramuscular and oral supplementation, with some studies suggesting oral administration as a potentially preferable option in certain contexts. Future research should include adequately powered comparative studies assessing patient-centered outcomes, such as symptom improvement, treatment adherence, and preference. Considerations of cost-effectiveness between sublingual and intramuscular approaches may also influence accessibility and real-world adoption, especially in resource-limited settings. This review was undertaken to establish a shared evidence base for a subsequent Delphi consensus on vitamin B12 replacement strategies, in which its findings were translated into graded clinical recommendations and a research agenda (Cohen et al., submitted).

## Contributors

RVC conceived and designed the review, provided clinical and methodological direction, and is the senior and corresponding author. DD and AFAS designed the search strategy, independently screened records, selected studies, extracted data, and assessed risk of bias, resolving disagreements by consensus, and conducted the descriptive synthesis. DD, AFAS, and RVC drafted the manuscript. JTC reviewed and edited the manuscript. DD and AFAS accessed and verified the data. RVC is the guarantor and had final responsibility for the decision to submit. All authors approved the final version.

## Data sharing statement

All studies included in this review are published and cited. The full search strategy, study-selection records, extracted data, and risk-of-bias assessments are available from the corresponding author on reasonable request.

## Declaration of interests

DD and AFAS are employees of HTAnalyze Consultoria e Treinamento Ltda, a consultancy providing health-technology-assessment and evidence-synthesis services, which received fees from Myralis Indústria Farmacêutica Ltda in connection with this review. JTC is an employee of Myralis Indústria Farmacêutica Ltda (Senior Manager, Strategy and Innovation), the manufacturer of a vitamin B12 product and the funder of this review. RVC reports research grants to his institution from Johnson & Johnson Medical Brazil and Medtronic Brazil, honoraria from Johnson & Johnson Medical Brazil, Medtronic, Novo Nordisk, Eli Lilly, and Currax, and participation on scientific advisory boards for Morphic Medical, Johnson & Johnson, and Novo Nordisk.
